# Visible Light Photochemical Reactions for Nucleic Acid-Based Technologies

**DOI:** 10.3390/molecules26030556

**Published:** 2021-01-21

**Authors:** Bonwoo Koo, Haneul Yoo, Ho Jeong Choi, Min Kim, Cheoljae Kim, Ki Tae Kim

**Affiliations:** Department of Chemistry, Chungbuk National University, Cheongju 28644, Korea; xlfhqk@gmail.com (B.K.); dbgksmf4@gmail.com (H.Y.); com5734@gmail.com (H.J.C.)

**Keywords:** nucleic acids, visible light, photochemical reactions, templated reaction, nucleic acid-based technologies

## Abstract

The expanding scope of chemical reactions applied to nucleic acids has diversified the design of nucleic acid-based technologies that are essential to medicinal chemistry and chemical biology. Among chemical reactions, visible light photochemical reaction is considered a promising tool that can be used for the manipulations of nucleic acids owing to its advantages, such as mild reaction conditions and ease of the reaction process. Of late, inspired by the development of visible light-absorbing molecules and photocatalysts, visible light-driven photochemical reactions have been used to conduct various molecular manipulations, such as the cleavage or ligation of nucleic acids and other molecules as well as the synthesis of functional molecules. In this review, we describe the recent developments (from 2010) in visible light photochemical reactions involving nucleic acids and their applications in the design of nucleic acid-based technologies including DNA photocleaving, DNA photoligation, nucleic acid sensors, the release of functional molecules, and DNA-encoded libraries.

## 1. Introduction

Ever since DNA was confirmed as genetic material in 1952 [1], nucleic acid, one of the central biomolecules, has been considered as a crucial molecule for understanding or manipulation of biological phenomena in living organisms. With this in mind, nucleic acid-based technologies have been designed for various biological purposes such as nucleic acid detection [2,3], amplification [4,5], sequencing [6], and ligation [7]. Although traditional biotechnologies mainly based on natural nucleic acids and enzymes are still widely used today (e.g., PCR), their design and functionality are very limited and thus efforts to develop novel nucleic acid-based technologies with additional functionality have been continuously demanded.

Nucleic acids have gained increasing attention from chemists as a functionalization target because of their pivotal role as the genetic backbone and their unique property of self-assembly programmed by sequence information. Over the past two decades, several chemical reactions involving nucleic acids have been explored to design nucleic acid-based technologies for various biological and therapeutic applications [8]. To develop these technologies, chemical reactions, such as chemical bond formation or cleavage of the (1) nucleic acid or (2) external molecules bound to nucleic acids have been studied [9]. These reactions have brought new concepts of molecular performance, which were not observed in canonical unmodified nucleic acid strands, and have resulted in remarkable improvements in nucleic acid-sensing, drug synthesis and release, nucleic acid nanotechnology, and molecular therapy [9,10,11,12,13,14,15]. In this context, the development of new bioorthogonal reactions for nucleic acids has a potential to accelerate the expansion of the scope of nucleic acid-based technologies.

To date, a large pool of chemical reactions has been adopted and introduced in the field of nucleic acids [16,17]. Among these, photochemical reactions have emerged as a potential tool for designing nucleic acid-based technologies owing to their advantages such as speed, a lack of the need for additional reagents, and a controllable reaction space [18]. After the discovery of UV-based DNA photoreactions reported in 1978 [19,20,21], nucleic acid photoreactions have been widely studied. Of late, the development of visible light-driven photocatalysis and the use of a ubiquitous light source such as sunlight has resulted in a paradigm shift in the reaction platform from UV light to visible light [22]. Visible light causes chemical reactions to proceed under extremely mild conditions, which is not the case with the use of UV light; thus, the reaction can be used for bioorthogonal labeling of nucleic acids and other biomolecules [23]. Moreover, the advent of the catalytic version of the visible light-driven reaction allows chemists to significantly diversify the reaction methodologies for nucleic acids and the functionality of nucleic acid-based technologies.

This review summarizes the recent developments (since 2010) in nucleic acid-based technologies, particularly visible light photochemical reactions. This review is not limited to nucleic acid-templated reactions of external molecules, but covers all reactions occurring in nucleic acids. Moreover, we discuss the major nucleic acid-based technologies that have recently been developed using the principle of visible light photochemical reactions. The reactions shown in this review are categorized based on the purpose of the reaction (types of nucleic acid-based technologies), which include cleavage or ligation of the nucleic acid strands and the synthesis and release of chemical compounds. We have thoroughly discussed the various reactions that nucleic acids undergo and described their properties and the improvements made by visible light photochemical reactions for nucleic acid-based technologies.

## 2. Nucleic Acid Strand Photocleavage

Techniques that lead to single- or double-strand DNA cleavage have been used as therapeutic strategies for inducing tumor cell death [24]. In this context, photocleavage of DNA has been widely used because of its advantages, such as low invasiveness and easy temporal and spatial control [25,26]. This technique, the so called photodynamic therapy (PDT), utilizes a photosensitizer (PS) that absorbs light and subsequently causes singlet oxygen or photoinduced electron transfer (PET), which could lead to oxidative damage and cleavage of DNA strands [27]. The recent developments in visible light-based photosensitizers relying on metal complexes (Ru and Ir) and organic compounds (riboflavin) have resulted in the design of more efficient and site-selective DNA photocleavage induced by the generation of reactive oxygen species.

### 2.1. Ru Complex-Based Methods

**Figure 1:**
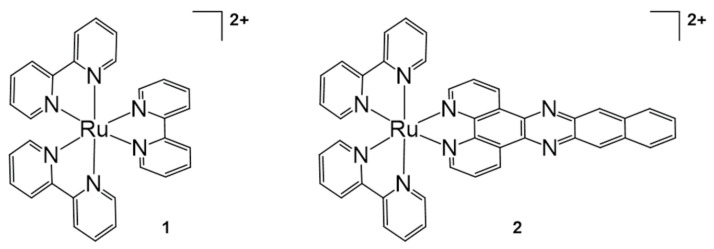
[Ru(bpy)_3_]^2+^ and [Ru(bpy)_2_(dppn)]^2+^ for DNA photocleavage. *Chem. Commun. (Camb.)* 2010, 46, 2426–2428, doi:10.1039/b925574e—Reproduced by permission of The Royal Society of Chemistry [30].

DNA photocleavage can be effectively performed using modified Ru(II) species in the presence of visible light irradiation based on ^1^O_2_ production by the Ru(II) complexes [28]. Since Ru(II) complexes showed a high population of triplet metal-to-ligand charge-transfer state (^3^MLCT) and ^1^O_2_ yields, they have been frequently utilized as agents for PDT using visible light [29]. The ligand change from the pristine tris(bipyridine)ruthenium(II) (**1**, [Ru(bpy)_3_]^2+^) is a versatile method for preparing modified Ru(II) complexes, and [Ru(bpy)_2_(dppn)]^2+^ (**2**, dppn = benzo[i]dipyrido[3,2-a:2′,3′-c]phenazine) was synthesized by Turro for DNA photocleavage in 2010 (Figure 1) [30]. The photophysical properties and π-stacking ability of [Ru(bpy)_2_(dppn)]^2+^ were investigated and compared with those of pristine [Ru(bpy)_3_]^2+^ using absorption spectra and ^1^H-NMR spectroscopy. Although the metal-based oxidation ability did not change, the ligand-based reduction potential was modified through dppn installation. In this system, complete DNA cleavage was achieved within 30 s under visible light irradiation (λ_irr_ ≥ 455 nm). It has been proposed that the combination of guanine oxidation and the production of reactive oxygen species was the main mechanism of action for DNA cleavage using [Ru(bpy)_2_(dppn)]^2+^.

**Figure 2:**
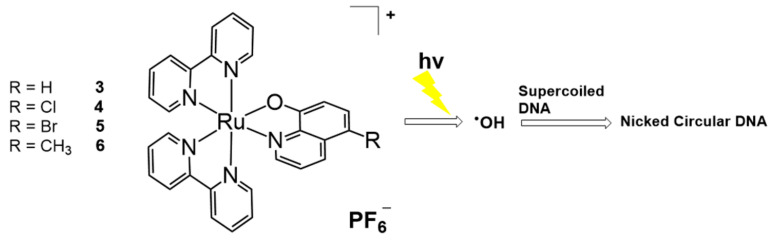
8-Quinolinol-based Ru complexes for DNA photocleavage. Adapted with permission from *Inorg. Chem.* 2016, 55, 4296–4300, doi:10.1021/acs.inorgchem.6b00028. Copyright (2021) American Chemical Society [31].

Molecular tuning with ligand exchange for [Ru(bpy)_3_]^2+^ was applied to the 8-quinolinol ligand for DNA photocleavage (Figure 2). Zhou and Wang synthesized [Ru(bpy)_2_(R-OQN)]^+^ (R-OQN = 5-chloro-8-oxyquinolate or 5-bromo-8-oxyquinolate) complexes, which could generate hydroxyl radicals and cleave DNA when irradiated with visible light [31]. The unique property of the 8-quinolinol ligand allowed for the high reducing ability and the generation of superoxide anions and hydroxyl radicals. In addition, the halogen effect on these non-innocent ligands was investigated. The 5-chloro- (**4**) and 5-bromo-substituted ligands (**5**) showed greater efficiency than the non-substituted 8-quinolinol (**3**) and 5-methyl-substituted ligands (**6**). With halogen-substituted [Ru(bpy)_2_(R-OQN)]^+^, metal-to-ligand charge transfer (MLCT) absorption was obtained at a maximum of 502–508 nm and could be considered a novel agent for photodynamic therapy (PDT) owing to its ability to absorb light at this wavelength.

**Figure 3:**
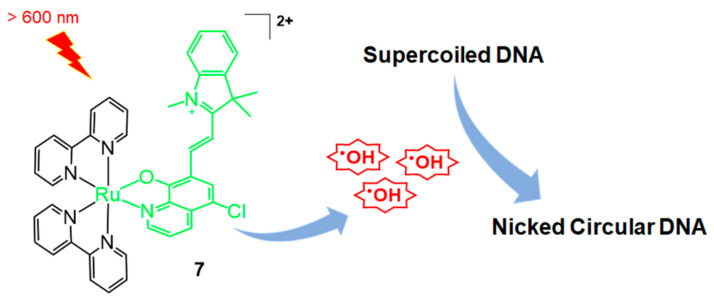
Merocyanine-functionalized Ru-bpy complex for DNA photocleavage. Adapted with permission from *Inorg. Chem.* 2017, 56, 1865–1873, doi:10.1021/acs.inorgchem.6b02459. Copyright (2021) American Chemical Society [32].

One year later, the same group reported enhanced Ru-bpy complexes for DNA photocleavage, which was based on the generation of hydroxyl radicals [32]. Using the 5-chloro-8-quinolate system, a merocyanine-functionalized ruthenium complex, [Ru(bpy)_2_(Cl-7-IVQ)]^2+^ (**7**, Cl-7-IVQ = 5-chloro-7-(2-(1,3,3-trimethyl-3*H*-indol-1-ium-2-yl)vinyl)quinolin-8-olate), was obtained and its DNA photocleavage ability under visible light irradiation was investigated (Figure 3). The highest efficiency was observed when the merocyanine group was introduced at the 7-position of quinolinol. Direct functionalization of the merocyanine group without the halogen moiety resulted in a compound with lower reactivity than that exhibited by [Ru(bpy)_2_(Cl-7-IVQ)]^2+^. Although the previous 5-chloro-8-quinolate-based [Ru(bpy)_2_(R-OQN)]^+^ showed the MLCT absorption maxima at wavelengths shorter than 550 nm, the present merocyanine system has intense adsorption in the 600–700 nm window. Moreover, the efficiency of hydroxyl radical generation was also increased for DNA photocleavage. Studies show that under red light irradiation, [Ru(bpy)_2_(Cl-7-IVQ)]^2+^ could selectively inactivate *Escherichia coli* bacterial cells over HeLa cells.

**Figure 4:**
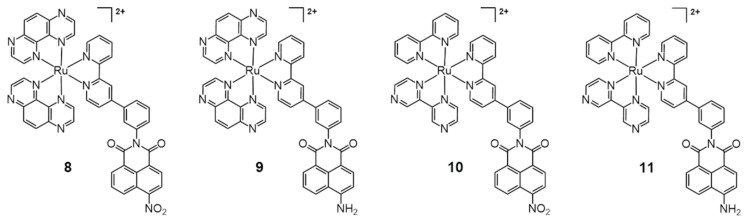
Ru complexes with TAP, bpy, and 1,8-naphthalimide-functionalized bpy for DNA photocleavage. Adapted with permission from *Inorg. Chem.* 2020, 59, 10874–10893, doi:10.1021/acs.inorgchem.0c01395. Copyright (2021) American Chemical Society [33].

In 2020, Elmes, Quinn, and Gunnlaugsson reported other modified Ru-bpy complexes for efficient DNA photocleavage [33]. 4-Nitro- and 4-amino-1,8-naphthalimide were previously used to form covalently bonded Ru-bpy complexes and studied for DNA photocleavage. In this system, highly efficient DNA cleavage was observed after irradiation for 5 min [34]. Additional molecular tuning was performed using the TAP (1,4,5,8-tetraazaphenanthrene) instead of the bpy ligand, and the substitution of 1,8-naphthalimide was changed from the para to the meta position (Figure 4, compounds **8**–**11**). The meta-arrangement allowed cleft formation and complementary DNA formation. Overall, TAP-based meta-arrangement with 1,8-naphthalimide-functionalized ruthenium complexes (**8**, **9**) showed enhanced DNA-photocleavage properties compared with Ru(bpy)-based 1,8-naphthalimide-functionalized complexes (**10**, **11**). In particular, compound **8** showed full conversion to the open form of the plasmid (1 mg/mL) within 30 min of irradiation.

**Figure 5:**
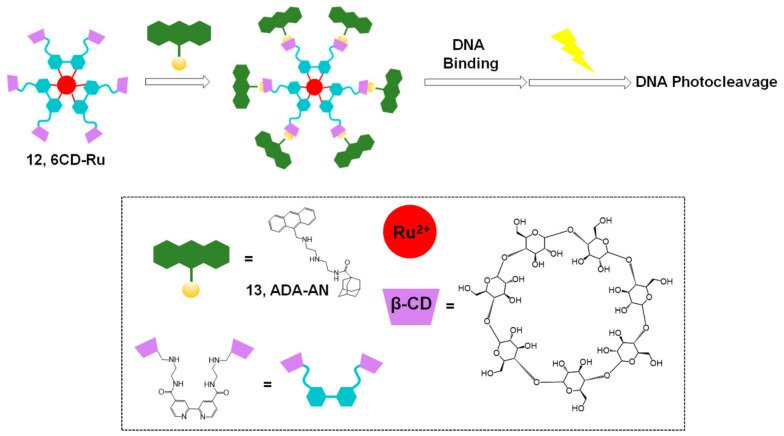
Photocleavage of DNA with 6CD-Ru and ADA-AN. Adapted with permission from *Bioconj. Chem.* 2018, 29, 1829–1833, doi:10.1021/acs.bioconjchem.8b00191. Copyright (2021) American Chemical Society [35].

In 2018, Liu and coworkers reported enhanced DNA binding and photocleavage abilities using a supramolecular strategy with a Ru(II) complex (Figure 5) [35]. PDT is considered an efficient and safe treatment for cancer, and several ruthenium complexes, including inorganic Ru(II), organometallic Ru(II) complexes, nanomaterial Ru(II), and Ru(II) polypyridine complexes, have been extensively studied for PDT [36]. In this system, a combination of hexa-β-cyclodextrin (CD)-appended Ru(II) complex (**12**, 6CD-Ru) and adamantane-modified anthracene (**13**, ADA-AN) was used for host–guest chemistry and the self-assembly to a supramolecular complex. This supramolecular complex was found to have non-covalent binding properties required for DNA photocleavage and enhanced efficiency owing to the six anthracene groups per Ru(II) complex. Moreover, the CD-based complex exhibited good water solubility. Therefore, efficient and enhanced photocleavage ability and antitumor activity of the Ru(II) complexes were achieved using the 6CD-Ru and ADA-AN systems under aqueous conditions with photoirradiation. These results suggest a new model that could be constructed for efficient PDT using visible light.

### 2.2. Methods Based on Other Photosensitizers

**Figure 6:**
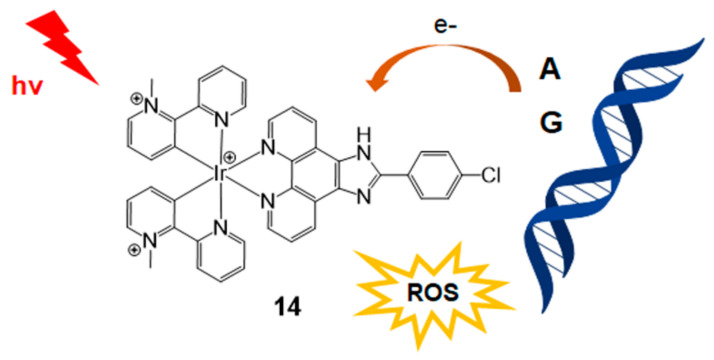
DNA photocleavage with bis-cyclometalated iridium (III) complex. Adapted with permission from *Inorg. Chem.* 2020, 59, 2426–2433, doi:10.1021/acs.inorgchem.9b03312. Copyright (2021) American Chemical Society [37].

The use of the iridium(III) complex for DNA photocleavage was reported by Defrancq and Elias in 2020 (Figure 6) [37]. This highly photooxidant iridium(III) complex is important for photoinduced electron transfer (PET) and DNA photocleavage by radical species containing purine DNA bases. In this study, CPIP (2-(4-chlorophenyl)-1*H*-imidazo[4,5-f] [1,10]-phenanthroline) was mainly used for iridium complexation. The CPIP ligand has been previously studied for selective G-quadruplex DNA recognition [38]. In addition, *N*-methyl pyridinium type 2,2′-C^N (1-methyl-[2,2′-bipyridin]-1-ium) was employed through cyclometalation to yield [Ir(2,2′-C^N)_2_CPIP]^3+^ (**14**). This bis-cyclometalated iridium(III) complex with guanine and adenine DNA base pairs triggered PET, which was confirmed using electrochemical experiments, luminescence quenching, and laser flash photolysis. This iridium(III) complex could be effectively used for DNA photocleavage through the photoproduction of H_2_O_2_, single oxygen, and PET with nucleobases.

**Figure 7:**
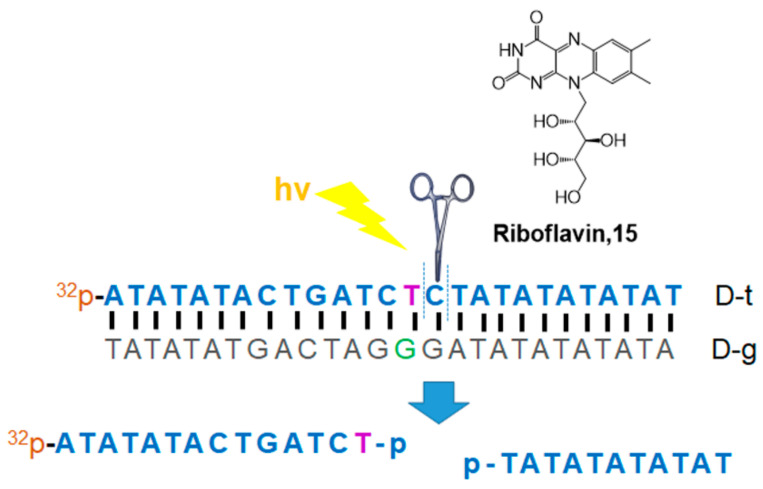
Selective DNA photocleavage of the G–T mismatch with riboflavin [39].

Selective DNA cleavage and tumor cell death were studied by Wang, Jiang, and Tang in 2017 using riboflavin (**15**, vitamin B2) irradiation (Figure 7) [39]. Riboflavin is an essential natural photosensitizer and a key molecule for redox chemistry in enzyme activity. Therefore, riboflavin is considered a promising anticancer agent for PDT. Based on their previous report showing sequence-specific photocleavage of RNA using riboflavin [40], Tang et al. revealed the recognition of riboflavin to G–T mismatch in DNA. It has been reported that several solid tumors are missing DNA mismatch repair (MMR) pathways; therefore, tumor cells generally have more G–T mismatches than normal cells in their DNA. In this system, riboflavin recognizes G–T base pairs, and singlet oxygen is generated through interaction with mismatched DNA for photocleavage of the neighboring nucleotides. This riboflavin system has several advantages for the photocleavage of DNA: (1) riboflavin is the most effective natural photosensitizer, (2) MMR deficiency can be solved in a short time, (3) low cytotoxicity, (4) essential nutrients for eukaryotic cell absorption, and (5) cell-specific activity could be monitored using this riboflavin system. Although the detailed mechanism of the riboflavin system has not been fully elucidated, it can provide an efficient PDT method for tumor cells. Types of photosensitizers for nucleic acid strand photocleavage and their properties are summarized in Table 1. Other compounds, such as naphthalimides, coronene, 2-quinolinium dicarbocyanine, and Fe(II)N4Py, were introduced to visible light-induced photocleavage of DNA strands [41,42,43,44,45,46].

## 3. Nucleic Acid Strand Photoligation

Photo-mediated DNA cross-linking is a powerful tool for the rapid and precise detection of nucleic acids, construction of nucleic acid nanostructures, and control of DNA functions [47,48]. Diverse photo-responsive oligonucleotides have been developed owing to their utility. Previously, nucleic acid strand cross-linking based on UV light was usually performed via the [2 + 2] photocycloaddition reaction of pyrimidines. For example, [2 + 2] photocycloaddition of pyrimidine and psoralen [49], 5-vinyldeoxyuridine [50], or 3-cyanovinylcarbazole [51,52,53] yielded a compound for constructing stable DNA nanostructures, and the reactions of 5-cyanovinyldeoxyuridine [54] or coumarin [55,56] were used as a probe for pyrimidine bases. In addition to [2 + 2] photocycloaddition, the self-reaction of p-stilbazole [57], anthracene moieties [18], and benzyl cation moieties [58,59] was also found to be applicable to DNA cross-linking. However, UV-A irradiation is generally required for the desired photochemical reaction, and this phototoxicity from UV radiation is a major concern in this field [60]. A safe light source, that is, visible light, is necessary for the biological application of photoinduced regulation.

### 3.1. Photo-Cross-Linking Based on [2 + 2] Photocycloaddition Reaction

**Figure 8:**
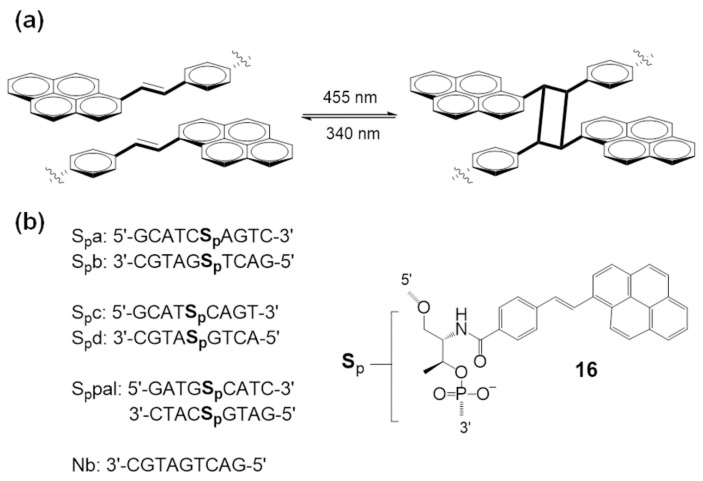
(**a**) Reversible [2 + 2] photocycloaddition of styrylpyrene. (**b**) Sequences of synthetic oligonucleotides and structure of **S_p_**. Reproduced from [61] by permission of John Wiley & Sons Ltd.

To overcome the limitations of systems based on UV irradiation, [2 + 2] photocycloadditions proceeding with visible light have been developed. In 2016, Asanuma et al. reported visible light-triggered photo-cross-linking of a DNA duplex via reversible [2 + 2] photocycloaddition using a styrylpyrene moiety (**16**, **S_p_**) [61]. This [2 + 2] photocycloaddition of styrylpyrene is well-established (Figure 8a).

Synthetic oligonucleotides were prepared with a styrylpyrene moiety, and photoinduced cross-linking was demonstrated using complementary oligonucleotides (Figure 8b). Under 455 nm light irradiation, the distinct absorption peak of styrylpyrene disappeared, and alkylpyrene peaks appeared at 338 nm and 354 nm. HPLC analysis and experimental results strongly support the occurrence of photo-cross-linking between styrylpyrenes from the oligonucleotide pair. Additionally, cycloreversion was studied under UV-A irradiation. Under 340 nm irradiation, the absorption at 390 nm was recovered, and 338 nm and 354 nm peaks disappeared. This is reported as the first example of visible-light-based photo-cross-linking that can be reversed by UV-A light.

**Figure 9:**
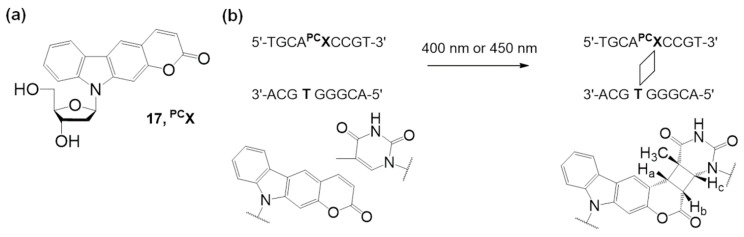
(**a**) Pyranocarbazole nucleoside **^PC^X**. (**b**) Photocycloaddition ([2 + 2]) between pyranocarbazole and pyrimidine. Adapted with permission from *Org. Lett.* 2018, 20, 2802–2805, doi:10.1021/acs.orglett.8b00593. Copyright (2021) American Chemical Society [62].

Fujimoto et al. developed a new DNA photo-cross-linking method that uses pyranocarbazole and visible light irradiation [62]. They designed a unique photo-cross-linking nucleoside bearing a pyranocarbazole moiety. They discovered a highly selective [2 + 2] cycloaddition between pyranocarbazole and pyrimidines in nucleic acids under 400 nm light irradiation (Figure 9).

Artificial oligonucleotides which have the pyranocarbazole nucleoside **^PC^X** (**17**) have demonstrated photo-cross-linking properties, and pyranocarbazole could form a [2 + 2] cycloadduct with its complementary strand of over 90% with thymine and 20% with cytosine under 400 nm light for 10 s.

**Figure 10:**
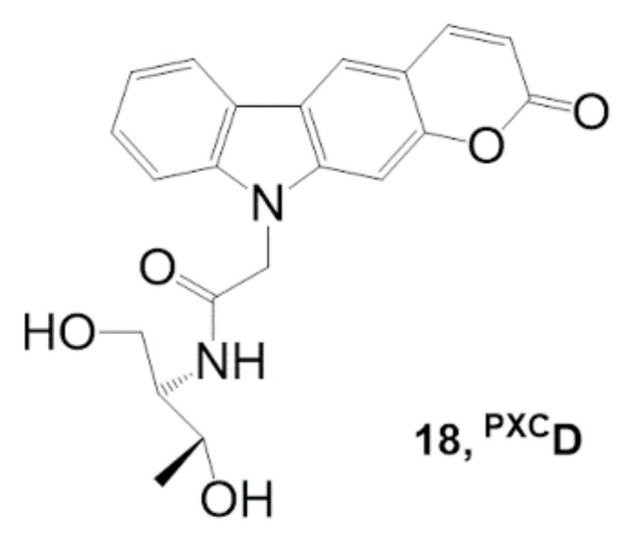
Alternative pyranocarbazole photo-cross-linker with _D_-threoninol **^PXC^D**. *RSC Adv.* 2019, 9, 30693–30697, doi:10.1039/c9ra06145b—Reproduced by permission of The Royal Society of Chemistry [63].

Fujimoto et al. modified their photo-cross-linking nucleoside to improve the cross-linking reactivity with cytosine [63]. They synthesized a new pyranocarbazole nucleoside **^PXC^D** (**18**) bearing a _D_-threoninol backbone instead of the 2′-deoxyribose backbone to accelerate the photo-cross-linking reaction with cytosine (Figure 10).

**^PXC^D** provided 4.3-fold improved reactivity with cytosine compared with **^PC^X**. The relatively flexible skeleton of _D_-threoninol may increase the accessibility of the pyrimidine moiety from complementary nucleotides. This photo-crosslinker is expected to be applicable to gene regulation in living cells.

### 3.2. Other Photo-Cross-Linking Methods

**Figure 11:**
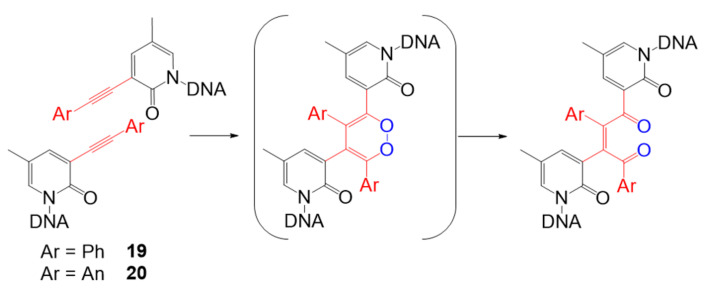
Design and structures for alkyne−alkyne photo-cross-linking based on base-flipping. Adapted with permission from *Org. Lett.* 2019, 21, 2833–2837, doi:10.1021/acs.orglett.9b00817. Copyright (2021) American Chemical Society [64].

Apart from advances in [2 + 2] photocycloaddition, other types of visible light photo-cross-linking methods have recently been designed. One of the representative works was performed by Onizuka et al. This group developed alkyne–alkyne photo-cross-linking based on base-flipping [64] to induce the flipping-out structure of oligonucleotides, 3-arylethynyl-5-methyl-2-pyridone nucleosides, which contain a phenyl (**19**) or an anthracenyl moiety (**20**) (Figure 11). In the base flipping-out field, two alkynes overlap with each other, and the [2 + 2 + 2] cycloaddition reaction proceed with the alkynes and molecular oxygen to form a 1,4-enedione structure through the single electron transfer (SET) mechanism. By using nucleosides containing an anthracenyl moiety, cross-linking products were readily obtained in 1 min using visible light at a wavelength of 440 nm.

Manicardi and coworkers developed a visible light-triggered oligonucleotide-templated reaction using furan-modified peptide nucleic acids [65]. Peptide nucleic acids are artificial nucleic acid mimics, and they have advantages with their electrically neutral backbone, such as lower electrostatic repulsion from DNA or RNA, naturally negatively charged phosphate backbones, and high stability under various experimental conditions. This group developed a furan oxidation-based interstrand cross-linking for nucleic acids [66] and expanded this methodology into peptide nucleic acid probes for DNA capture [67] and visible light-triggered peptide labeling [68,69].

**Figure 12:**
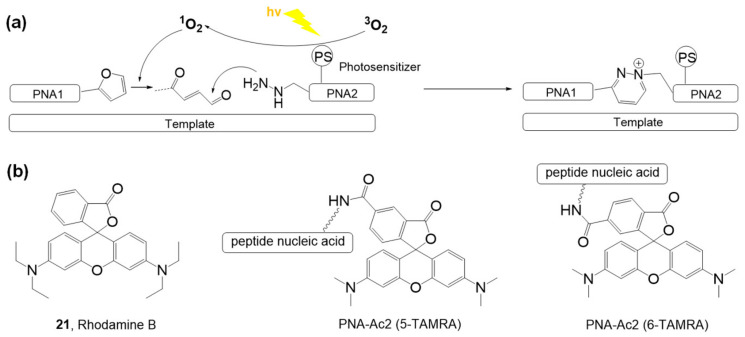
(**a**) Photo-triggered ligation of PNA strands by utilizing photoexcited singlet oxygen. (**b**) Structure of rhodamine B (photosensitizer) and photosensitizer-containing nucleotides. *Chem. Sci.* 2020, 11, 11729–11739, doi:10.1039/d0sc04875e—Reproduced by permission of The Royal Society of Chemistry [65].

The photoinduced ligations based on the reaction between γ-keto-enal and a nucleophile were demonstrated with furan-bearing oligopeptide nucleic acids and a photosensitizer, rhodamine B (**21**), under white light (100 W halogen lamp) irradiation (Figure 12a). The ligation occurred with matched complementary DNA or RNA sequences, and the reaction was not observed in the case of mismatched complementary nucleotides. When the photosensitizer moiety (Figure 12b, 5-TAMRA or 6-TAMRA) was included in oligopeptide nucleic acids bearing nitrogen nucleophiles, the ligation efficiency increased. This phenomenon could be explained by the interaction between negatively charged DNA and positively charged rhodamine B. Free rhodamine B easily interacts with DNA, decreasing the efficiency of singlet oxygen generation. However, a fixed photosensitizer on an oligopeptide chain can fully function to provide singlet oxygen in the reaction. Surface-templated oligopeptides also showed good ligation properties, which may be applied to high-throughput systems for efficient analysis of short nucleotides.

## 4. Chemical Bond Photocleavage of Nucleic Acids

Photochemical bond cleavage of nucleic acids can be used for the conditional release of functional molecules only in the presence of particular target nucleic acid sequences. For the reaction systems, two probe oligonucleotides that bind to target sequences are required: one has a photosensitizer and the other generally contains a fluorophore with a cleavable linker. Such systems are generally applied to release fluorophores or bioactive molecules for biological and therapeutic applications, such as nucleic acid sensing [10,70] and drug release [9]. In addition to photochemical reactions activated upon UV-A irradiation [71], inspired by the development of new photosensitizer-cleavable linker pairs, various visible light-driven photoredox catalytic reactions have recently been used for the conditional release of functional molecules. Major achievements were made by two groups.

### 4.1. Singlet Oxygen-Mediated Fluorogenic Reaction

**Figure 13:**
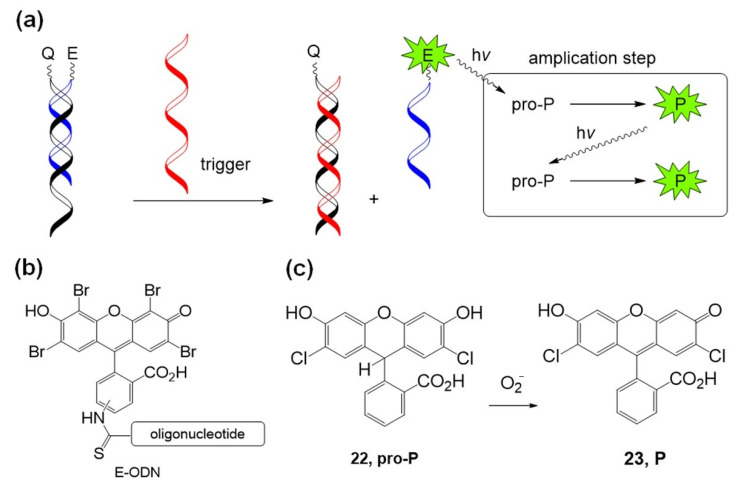
(**a**) Concept of the autocatalytic photochemical reaction. (**b**) Structure of eosin–oligonucleotide. (c) Structure of pro-P and P. *Chem. Commun. (Camb.)* 2011, 47, 1243–1245, doi:10.1039/c0cc02508a—Reproduced by permission of The Royal Society of Chemistry [72].

Mokhir et al. developed several fluorogenic photochemical reactions using molecular oxygen activation. In 2011, an autocatalytic photochemical reaction was developed using an eosin photosensitizer containing an oligonucleotide and a pro-photosensitizer [72]. Figure 13 shows the autocatalytic photochemical reaction. First, eosin–oligonucleotide E–ODN was combined with a complementary quencher containing oligonucleotide Q–ODN. In the presence of a trigger oligonucleotide that has higher affinity with quencher–oligonucleotide Q–ODN than with photosensitizer–oligonucleotide E–ODN, free eosin–oligonucleotide E–ODN is released and starts its photochemical reaction under green light irradiation. Photoexcited eosin generates singlet oxygen (^1^O_2_) and superoxide anion radical (O_2_^•−^), and the anion radical drives the autocatalytic photochemical oxidation of pro-photosensitizer pro-P (**22**, pro-2′,7′-dichlorofluorescin) to produce colored and fluorescent products, P (**23**).

**Figure 14:**
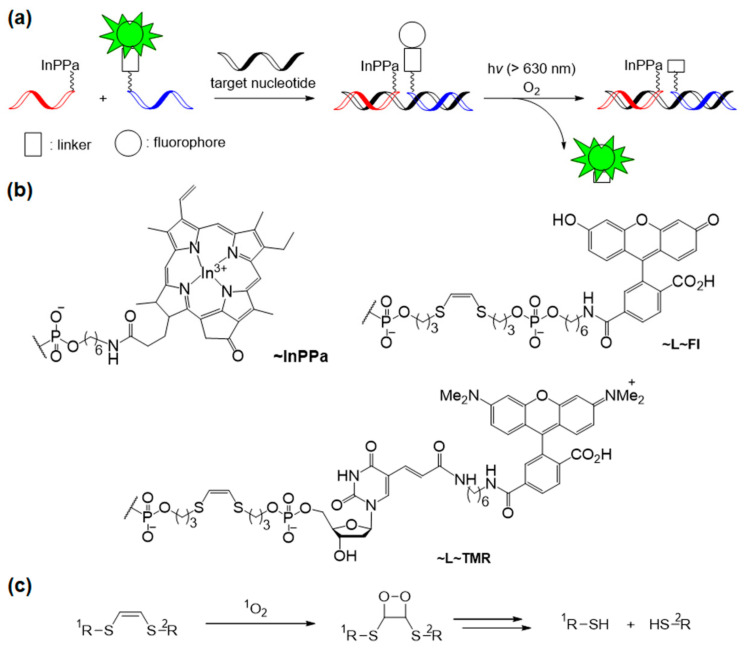
(**a**) Concept of the photochemical reaction-based fluorogenic nucleic acid probe. (**b**) Structures of photosensitizer probe and fluorophore probes. (**c**) Mechanism of -SCH=CHS- linker cleavage by ^1^O_2_. *Chem. Commun. (Camb.)* 2012, 48, 9664–9666, doi:10.1039/c2cc33827k—Reproduced by permission of The Royal Society of Chemistry [73].

In 2012, Mokhir et al. reported a new fluorogenic probe for specific nucleic acid templates by releasing fluorescent dyes under safe red light (635 nm) irradiation [73]. One probe has a photosensitizer located at the 3′-terminus of the oligonucleotide backbone. The other oligonucleotide probe contains a fluorophore linked with a cleavable linker (L~Fl or L-TMR) at the 5′-terminus (Figure 14a,b). This system uses In^3+^(pyropheophorbide-a) chloride (InPPa) as a photosensitizer and ^1^O_2_-sensitive -SCH=CHS- linker to cleave the fluorophore and oligonucleotide backbone (Figure 14c).

Compared with the free fluorescein Fl fluorophore, the fluorescein-linked oligonucleotide Fl~L-ODN showed a 5.4 times lower fluorescence intensity at 520 nm than free Fl. This quenching is caused by the interaction between the Fl dye and the combined oligonucleotide. Moreover, the nucleotide complex with the template and Fl~L-ODN further quenched the emission of Fl by a factor of 10.7. In the presence of a target nucleotide template, the linker readily accepts ^1^O_2_ because of its proximity to the ^1^O_2_ generator, i.e., the photosensitizer; therefore, bright emission produces the acceleration of the released free fluorophore. This method worked at a target concentration of 100 nM and was thought to be a useful tool for the quantitative visualization of nucleic acids and the simultaneous detection of multiple RNA targets.

**Figure 15:**
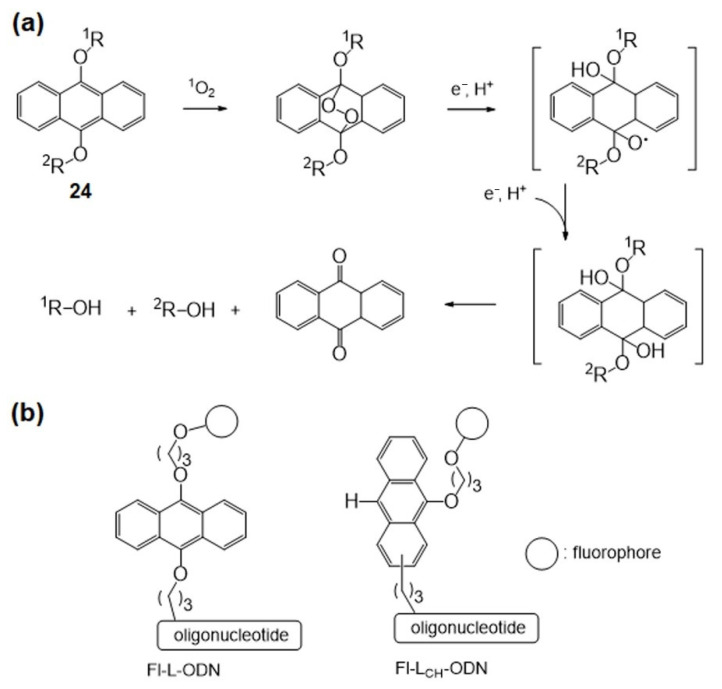
(**a**) The mechanism of 9,10-dialkoxyanthracene linker cleavage by ^1^O_2_. (**b**) Structures of fluorophore-linked oligonucleotides. Adapted with permission from *Bioconj. Chem.* 2019, 30, 2023–2031, doi:10.1021/acs.bioconjchem.9b00299. Copyright (2021) American Chemical Society [74].

The Mokhir group developed a new red light-triggered fluorogenic reaction with picomolar sensitivity in 2019 [74]. They initially took advantage of the highly ^1^O_2_-sensitive 9,10-dialkoxyanthracene moiety (**24**) instead of -SCH=CHS- used in the previous work as a linker between the fluorophore and the probe backbone [75] (Figure 15).

With the 9,10-dialkoxyanthracene linker, cleavage requires external reductants, which means that the reaction is a second-order process (Figure 15a). The concentration of the complex of probes and templated nucleotides is practically less than 2 nM, which is a limitation of the Fl~L-ODN system. To solve this kinetic reaction problem, fluorophore-linked nucleotides, including an intramolecular electron donation moiety, can change the reaction rate to the first-order instead of the second-order process, which could increase the sensitivity of the photo-triggered nucleotide-templated reaction. The mono-9-alkoxyanthracene-linked oligonucleotide Fl-L_CH_-ODN provides 2e^−^ via oxidation of the 10-C-H moiety (Figure 15b). As a result, Fl-L_CH_-ODN undergoes a faster reaction than Fl~L-ODN. To increase the signal-to-noise ratio, background fluorescence must be minimized. The 1,4-diamino-9,10-anthraquinone moiety is an efficient quencher of photosensitizers and fluorophores, and it is stable under photochemical reaction conditions. The addition of quencher nucleotides Q–ODNs suppresses the background emission to improve the signal-to-noise ratio by over 1000-fold, and this red light-triggered fluorogenic templated reaction allows detection of ~10 pM nucleotide-templated sequences in the solution.

### 4.2. Ru-Catalyzed Photoreduction

**Figure 16:**
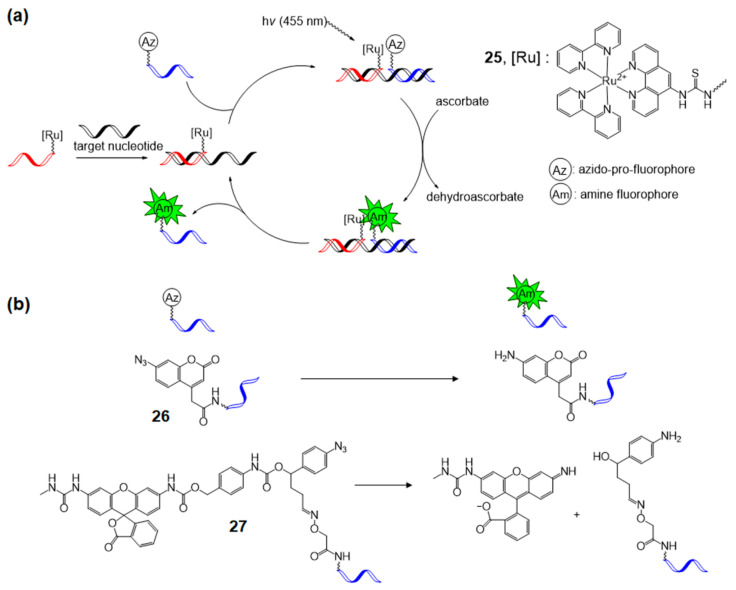
(**a**) Concept of the Ru-catalyzed photoreduction of the nucleic acid-templated fluorophore uncaging reaction. (**b**) The structure of azido-pro-fluorophores and active fluorophores. Adapted with permission from *Org. Lett.* 2012, 14, 482–485, doi:10.1021/ol203029t. Copyright (2021) American Chemical Society [76].

Winssinger et al. focused on the Ru-catalyzed photoreduction of nucleic acid-templated fluorophore uncaging reactions. In 2012, the first nucleotide-templated photoreduction of an azido-pro-fluorophore probe was performed using a catalytic Ru-containing peptide nucleic acid probe [76,77]. Azido-pro-fluorophore, azido-coumarin (**26**), or azido-rhodamine (**27**) was used, and the reduction toward amine was required to turn on the fluorophore function. The Staudinger reaction is a well-known process for the reaction of azide into amines, and several biological applications have been reported [78,79]. However, the Staudinger reaction requires excess phosphine probe due to phosphine’s sensitivity to oxidation. Photocatalytic reduction of azide with [Ru(bpy)_3_]^2+^ in the presence of a stoichiometric reductant, for example, ascorbate or NADPH (nicotinamide adenine dinucleotide phosphate), was developed in 2011 by Liu et al. [80] and this methodology was applied to nucleic acid-templated reactions (Figure 16a). [Ru(bpy)_2_phen]^2+^-tethered photosensitizer probe was synthesized from the commercially available [Ru(bpy)_2_(5-NCSphen)]^2+^ complex (**25**). The desired photoreaction occurred with a 2% loading of the Ru-photosensitizer probe under 455 nm light irradiation on a 10 nM matching complimentary template.

**Figure 17:**
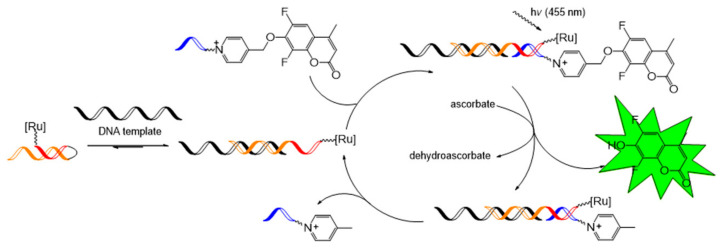
Concept of the Ru-catalyzed photoreduction of the nucleic acid templated with a pyridinium linker. Adapted with permission from *J. Am. Chem. Soc.* 2017, 139, 1444–1447, doi:10.1021/jacs.6b12764. Copyright (2021) American Chemical Society [81].

For signal amplification, a highly cleavable pyridinium linker was used together with a Ru-photocatalyst to release a coumarin fluorophore as a fluorescent signal [81] (Figure 17). The pseudo-first-order rate, *k*_app_, of these templated reactions was observed as 138 × 10^−3^/s and is known as the fastest templated reaction rate. With this reaction in hand, to gain turnover efficiency and vigorous emission intensity in the catalytic photofluorogenic reaction, the templated reaction system was tested by changing the lengths of peptide nucleic acid probes (4–9 mer). As a result, the 5-mer peptide nucleic acid pair showed the most efficient turnover with the 2% Ru-photosensitizer probe, while shorter or longer peptide nucleic acid probes had a lower turnover frequency. Based on this, a hairpin sensing system was designed for nucleic acid detection, and this system achieved a high turnover frequency of over 100 h^−1^ using 0.1% template loading.

Inspired by this remarkable achievement, a pair of Ru-photocatalysts and pyridinium linkers has been further utilized to design various nucleic acid-sensing techniques for the detection of miRNA [82,83], double-stranded RNA [84], and single nucleotide polymorphism (SNP) [85].

## 5. Chemical Bond Formation in Nucleic Acids

Nucleic acid strands are hybridized with complementary sequences with extremely high stability. Chemical reactions leveraged by such unique features of nucleic acids have enabled a platform for combinatorial synthesis based on sequence-directed chemical bond formation. This DNA-encoded library technology (DELT) has been utilized to prepare and screen a large set of diverse small molecule drug candidates that specifically bind to target biomolecules present in the pharmaceutical industry [86,87,88,89]. Particularly, a DNA-encoded library based on the split and pool approach (combinatorial synthesis with repeating reaction cycles: split, reaction, and DNA tagging, mix steps) enables preparation of 10^6^ to 10^12^ different molecules [90,91]. Introduction of visible light-based photochemical reactions into the DELT allows medicinal chemists to expand the reaction scope and diversify small molecule drugs’ chemical structures.

### 5.1. Ir-Based Photocatalytic Reactions

**Figure 18:**
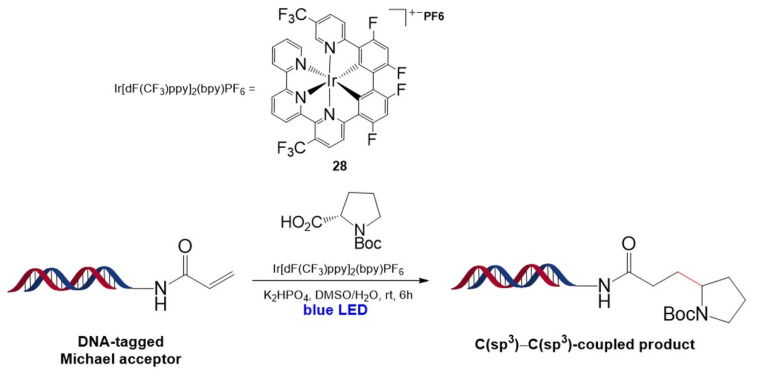
On-DNA decarboxylative alkylation of α-amino acids using an Ir-based photocatalyst. Reproduced from [93] by permission of John Wiley & Sons Ltd.

In 2018, inspired by a report of a Giese-type reaction discovered by MacMillan et al. [92], the Ir photocatalyst was first embedded in DNA strands by Flanagan et al. [93]. In this reaction, α-amino radicals were generated by Ir[dF(CF_3_)ppy]_2_(bpy)PF_6_ (**28**) from the decarboxylation of the carboxyl radical and reacted with the DNA-tagged Michael acceptor to produce the C(sp^3^)–C(sp^3^)-coupled product, γ-amino carboxamide (Figure 18). The reaction worked well over a large scope of α-amino acids, including N-Boc- or Cbz-protected α-amino acids and heterocyclic α-amino acids, and was tolerated by most functional groups such as alcohols, carboxylic acids, carboxamides, and guanidines. The reaction has potential application in a three-cycle DNA-encoded library technique comprising DNA–radical acceptor conjugation via acylation, decarboxylative alkylation, and capping of the deprotected free amine, which may enable screening of a library of 750,000,000 compounds (100 radical acceptors × 500 amino acids × 1500 capping agents).

**Figure 19:**
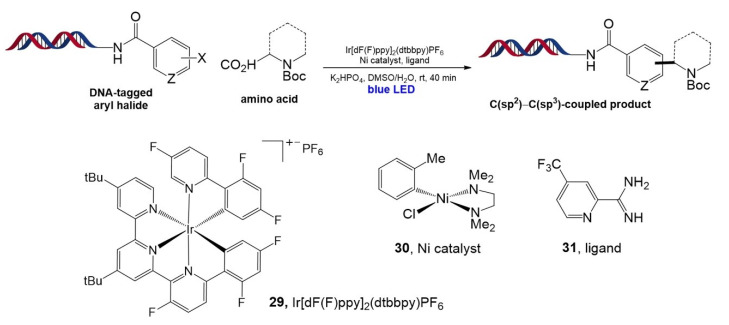
Decarboxylative arylation of amino acids with DNA-tagged aryl halides using dual catalysis. Adapted with permission from *ACS Comb. Sci.* 2019, 21, 588–597, doi:10.1021/acscombsci.9b00076. Copyright (2021) American Chemical Society [94].

In 2019, water- and DNA-compatible decarboxylative arylation using α-amino acids and aryl halides conjugated with DNA was established by the same group [94]. In this work, dual catalysis using Ir and Ni(0) invented by MacMillan and Doyle [95] was introduced onto DELT DNA strands. The optimized reaction was observed when the Ir photocatalyst (**29**, Ir[dF(F)ppy]_2_(dtbbpy)PF_6_), air-stable nickel pre-catalyst (**30**), and electron-deficient pyridyl carboxamidine ligand (**31**) were used (Figure 19). The reaction proceeded well with various α-amino acids (N-BOC-protected α-amino acids, cyclic and bicyclic amino acids) and aryl halide pairs with moderate to high yields. Finally, the potential reaction for the DELT was successfully demonstrated by parallel synthesis performed in a 96-well plate with a blue LED array.

Moreover, in 2020, Berst and coworkers implemented a dual-catalytic metallaphotoredox reaction on DNA for decarboxylative cross-coupling of carboxylic acids to aryl halides tagged with DNA under anhydrous conditions for the synthesis of a DNA-encoded library [96].

**Figure 20:**
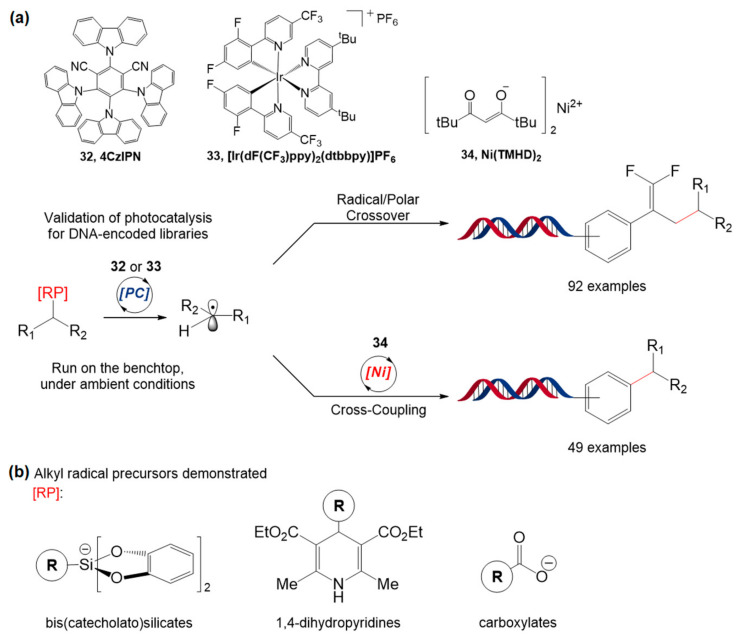
(**a**) DNA-encoded library synthesis based on cross-coupling based on Ni/photoredox dual catalysis and photoredox-catalyzed radical/polar crossover. (**b**) Structure of alkyl radical precursors in this study. Adapted with permission from *J. Am. Chem. Soc.* 2019, 141, 3723−3732, doi:10.1021/jacs.9b00669. Copyright (2021) American Chemical Society [97].

In the same year, Molander et al. demonstrated two reaction protocols based on visible light for the DELT: C(sp^2^)−C(sp^3^) cross-coupling based on Ni/photoredox dual catalysis and photoredox-catalyzed radical/polar crossover (Figure 20a) [97]. For cross-coupling, 4-alkyl-1,4-dihydropyridines (DHPs) and amino acids were used as radical precursors, and the organic dye 1,2,3,5-tetrakis(carbazol-9-yl)-4,6-dicyanobenzene (**32**, 4CzIPN) showed the best results with DHP, whereas the Ir catalyst, [Ir(dF(CF_3_)ppy)_2_(dtbbpy)]PF_6_ (**33**), showed increased product conversion when N-BOC amino acids were used. They also demonstrated the formation of gem-difluoroalkenes through the defluorinative alkylation of trifluoromethyl alkenes based on radical/polar crossover chemistry. For this reaction, alkyl bis(catecholato)silicates, DHPs, and amino acids were tested as water-compatible radical precursors (Figure 20b); the silicate and DHP worked well with 4CzIPN. Importantly, these two protocols can proceed under ambient conditions within minutes and are compatible with functional group changes, water, air, and DNA strands, which facilitates the design of the encoded library synthesis.

**Figure 21:**
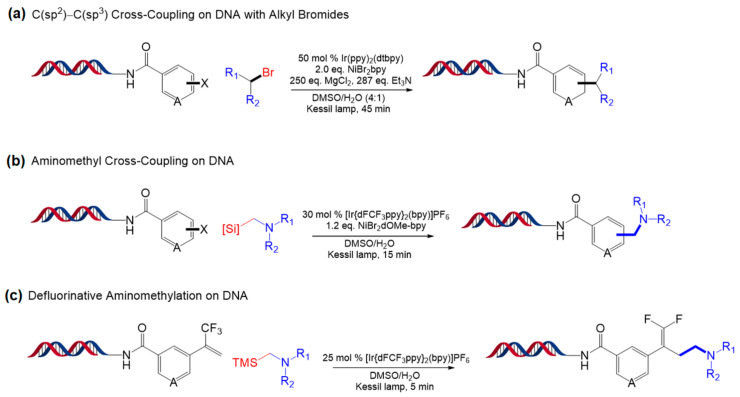
(**a**) Reductive C(sp^2^)−C(sp^3^) coupling of primary or secondary alkyl bromides to DNA-tagged aryl halides. (**b**) Aminomethyl cross-coupling of (hetero)aryl halide and α-silylamines. (**c**) Radical/polar crossover defluorinative alkylation using α-silylamines. Adapted with permission from *Org. Lett.* 2020, 22, 1046–1051, doi:10.1021/acs.orglett.9b04568. Copyright (2021) American Chemical Society [98].

One year later, Molander and coworkers developed three approaches to expand the reaction scope of Ni/photoredox that uses α-amino acids and DHP [98]. The first approach is reductive C(sp^2^)−C(sp^3^) coupling of primary or secondary alkyl bromides to DNA-tagged aryl halides (bromides and iodides), and the best result was obtained with [Ir(dtbbpy)(ppy)_2_]PF_6_ (Figure 21a). Using the same catalyst, aminomethyl cross-coupling of (hetero)aryl halide and α-silylamines was performed on DNA as the second approach. Alpha-silylamines are thought to be oxidized by [Ir[dFCF_3_ppy]_2_(bpy)]PF_6_ to yield α-aminomethyl radicals, which led to a coupled reaction mediated by the nickel catalyst (Figure 21b). Finally, α-silylamines were also applied to radical/polar crossover defluorinative alkylation (Figure 21c). All three approaches used in the study showed reaction completion within minutes and had high functional group tolerance. These reactions are expected to enable derivatization of diverse chemical compounds for a large number of DNA-encoded libraries.

**Figure 22:**
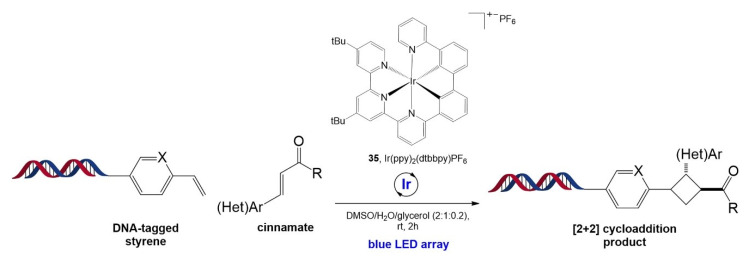
Reaction of [2 + 2] cycloaddition catalyzed by iridium-based photocatalyst Ir(ppy)_2_(dtbbpy)PF_6_ for DNA-encoded library synthesis. Adapted with permission from *Org. Lett.* 2020, 22, 2908–2913, doi:10.1021/acs.orglett.0c00574. Copyright (2021) American Chemical Society [101].

Very recently, inspired by previous studies regarding [2 + 2] cycloaddition [99,100], Kӧlmel and co-workers developed a [2 + 2] cycloaddition reaction for cyclobutane formation catalyzed by an iridium-based photocatalyst, Ir(ppy)_2_(dtbbpy)PF_6_ (**35**) (Figure 22) [101]. The cycloaddition reaction was carried out using a DNA-tagged styrene and a cinnamate substrate. The mechanism involves the formation of a cinnamate triplet state arising from triplet energy transfer from the Ir photocatalyst to the cinnamate, followed by an intramolecular radical−radical combination of the 1,4-diradical intermediate generated from the formation of the C(sp^3^)−C(sp^3^) bond between the cinnamate triplet state and the styrene. The reaction is well-tolerated by the variation in substituents on the aromatic ring, types of carboxylic derivatives of the enone substrate, and types of styrene derivatives, demonstrating excellent functional group tolerance. This [2 + 2] cycloaddition was further applied to prepare three-cycle DNA-encoded libraries containing a unique cyclobutane ring in the middle, achieved by three-step functionalization: amide coupling, [2 + 2] cycloaddition, and amide coupling or reductive amination.

### 5.2. Other Photocatalytic Reactions

**Figure 23:**
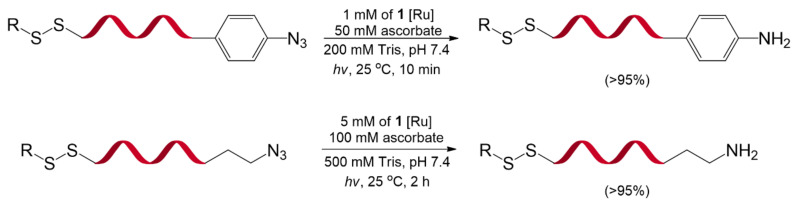
Ru(II)-catalyzed azide reduction on the DNA strand. Reprinted by permission from Macmillan Publishers Ltd.: *Nat. Chem.* 2011, 3, 146–153, doi:10.1038/nchem.932. Copyright (2021) [80].

Azide reduction catalyzed by Ru(II) complexes (**1**) and visible light was first developed by Liu et al. in 2011 [80] (Figure 23). The reaction showed high chemoselectivity over many functional groups, including alcohols, phenols, acids, alkenes, alkynes, aldehydes, alkyl halides, alkyl mesylates, and disulfides. Moreover, owing to its mild conditions and biocompatibility, this reaction was introduced into nucleic acids, protein enzymes, and oligosaccharides. Such remarkable properties of the reaction could potentially be used for the DELT.

**Figure 24:**
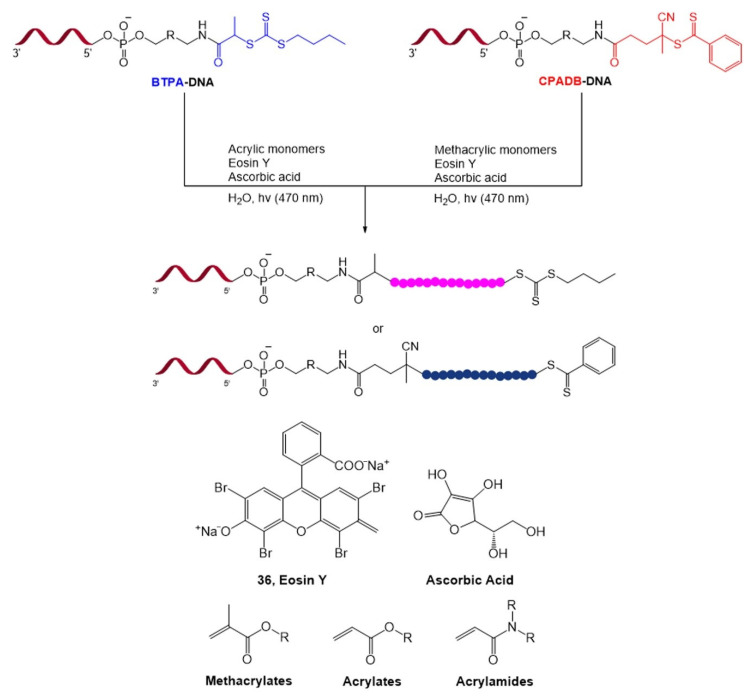
Visible light-based photopolymerization of methacrylates, acrylates, or acrylamide monomers on a DNA strand. Adapted with permission from *Biomacromolecules* 2019, 20, 212–221, doi:10.1021/acs.biomac.8b01328. Copyright (2021) American Chemical Society [102].

In addition to the DELT, a visible light photochemical reaction was used for the construction of DNA−polymer conjugates [102]. To overcome the low yield of the “grafting to” approach in which a presynthesized polymer is conjugated with DNA, the “grafting from” approach based on blue light-mediated reversible addition–fragmentation chain transfer (Photo-RAFT) polymerization has been developed [103]. In this reaction, an organic photocatalyst, Eosin Y (**36**), was employed to generate hydrogen peroxide from oxygen. Subsequently, ascorbic acid reduced hydrogen peroxide to produce radical species that can initiate polymerization. Methacrylates, acrylates, or acrylamide monomers were polymerized from 4-cyano-4-(phenylcarbonothioylthio)pentanoic acid (CPADB), or 2-[butylthio)carbonothioyl]propionic acid (BTPA), and DNA–polymer conjugates with molecular weights above 30,000 g mol^−1^ were achieved (Figure 24). The RAFT approach based on biocompatible dyes is a promising polymerization platform for constructing DNA–polymer conjugates in biological environments.

## 6. Conclusions and Outlook

In this review, the visible light photochemical reactions occurring on nucleic acids and their biological applications were explored. Owing to various advantageous properties such as extremely mild condition requirements, easy spatiotemporal control, and lack of extra additives, visible light-driven reactions have enabled chemists to design novel biological systems with high biocompatibility. They have brought significant improvements to biotechnology, especially for nucleic acid-based technologies. The recent achievements of on-nucleic acid photochemical reactions are categorized by their manipulation patterns: chemical bond cleavage and formation to manipulate nucleic acids or external molecules bound to nucleic acids.

First, photosensitizer reactions that can generate reactive oxygen species are widely introduced into nucleic acids for cancer therapy and photodynamic therapy (PDT) technologies. To date, novel designs of photosensitizers with extended and new ligand structures have been studied for more efficient nucleic acid cleavage with a broader range of irradiation wavelengths [43]. Such development also facilitated the design of sequence-specific photocleavage and highly efficient photocleavage systems based on supramolecular complexes.

Second, the visible light photochemical reaction has also been an efficient tool for connecting nucleic acid strands to hold their structure under milder conditions. This reaction is usually applied to nucleic acid techniques to regulate DNA function and the construction of DNA nanostructures. For these applications, [2 + 2] photocycloaddition has been extensively used to date; however, chemical transformation based on visible light has emerged as a versatile tool for ligation of nucleic acid strands.

Third, a variety of visible light photoredox catalysis reactions have brought outstanding improvements to the release of functional molecules, which can be applied to nucleic acid-sensing techniques. Ruthenium photocatalysts and other visible light photosensitizers are introduced into templated reactions and enable higher signal amplification and more sensitive nucleic acid-sensing. Many studies regarding the optimization of chemical reaction pairs and systematic design of templated reactions are currently being performed to detect nucleic acids existing in low abundance.

Finally, chemical transformation arising from visible light was carried out to prepare DNA-encoded library technologies. Various transition metal catalysts, including Ru, Ir, and Ni, have been widely exploited for C–C bond formation under visible light irradiation to build diverse chemical structures, which are beneficial for finding novel pharmaceutical compounds. Such visible light photoredox catalysis on DNA enabled not only mild synthesis, which makes it DNA- and functional group-compatible, but also diverse molecular transformations under aqueous conditions. Moreover, efforts to make photoredox catalysis more tolerant to other functional groups and available in ambient conditions (e.g., open air) have been made for the reliable and rapid preparation of a large pool of chemical libraries.

Leveraged by tremendous ongoing developments in new visible light photochemical reactions [104,105,106], these nucleic acid-based techniques have also experienced a paradigm shift from classic photochemical reactions to photocatalysis, providing greater efficiency and expanding the reaction scope. Inspired by such improvements, novel nucleic acid-based techniques, which are more biocompatible, chemoselective, less restricted in terms of required conditions, faster, more user-friendly, and cost-effective are expected to be developed for biotechnology and chemical biology in the future.

## Figures and Tables

**Table 1:** Types of photosensitizers for nucleic acid strand photocleavage and their properties.

Photosensitizer	Ligands	Properties	References
Ru complex (Figure 5)	ADA-AN	- PDT with visible light - Water-soluble	[35]
Ir complex (Figure 6)	CPIP	- G–A DNA base pair triggered PET	[37]
Riboflavin (Figure 7)		- Selective DNA photocleavage of the G–T mismatch	[39]

## Data Availability

No new data were created or analyzed in this study. Data sharing is not applicable to this article.
